# Sensing Technology to Monitor Behavioral and Psychological Symptoms and to Assess Treatment Response in People With Dementia. A Systematic Review

**DOI:** 10.3389/fphar.2019.01699

**Published:** 2020-02-04

**Authors:** Bettina S. Husebo, Hannah L. Heintz, Line I. Berge, Praise Owoyemi, Aniqa T. Rahman, Ipsit V. Vahia

**Affiliations:** ^1^ Department of Global Public Health and Primary Care, Centre for Elderly and Nursing Home Medicine, University of Bergen, Bergen, Norway; ^2^ Department of Nursing Home Medicine, Municipality of Bergen, Bergen, Norway; ^3^ Division of Geriatric Psychiatry, McLean Hospital, Belmont, MA, United States; ^4^ NKS Olaviken Gerontopsychiatric Hospital, Bergen, Norway; ^5^ Department of Psychiatry, Harvard Medical School, Boston, MA, United States

**Keywords:** dementia, sensoring, monitoring, behavior, therapy

## Abstract

**Background:**

The prevalence of dementia is expected to rapidly increase in the next decades, warranting innovative solutions improving diagnostics, monitoring and resource utilization to facilitate smart housing and living in the nursing home. This systematic review presents a synthesis of research on sensing technology to assess behavioral and psychological symptoms and to monitor treatment response in people with dementia.

**Methods:**

The literature search included medical peer-reviewed English language publications indexed in Embase, Medline, Cochrane library and Web of Sciences, published up to the 5^th^ of April 2019. Keywords included MESH terms and phrases synonymous with “dementia”, “sensor”, “patient”, “monitoring”, “behavior”, and “therapy”. Studies applying both cross sectional and prospective designs, either as randomized controlled trials, cohort studies, and case-control studies were included. The study was registered in PROSPERO 3^rd^ of May 2019.

**Results:**

A total of 1,337 potential publications were identified in the search, of which 34 were included in this review after the systematic exclusion process. Studies were classified according to the type of technology used, as (1) wearable sensors, (2) non-wearable motion sensor technologies, and (3) assistive technologies/smart home technologies. Half of the studies investigated how temporarily dense data on motion can be utilized as a proxy for behavior, indicating high validity of using motion data to monitor behavior such as sleep disturbances, agitation and wandering. Further, up to half of the studies represented proof of concept, acceptability and/or feasibility testing. Overall, the technology was regarded as non-intrusive and well accepted.

**Conclusions:**

Targeted clinical application of specific technologies is poised to revolutionize precision care in dementia as these technologies may be used both by patients and caregivers, and at a systems level to provide safe and effective care. To highlight awareness of legal regulations, data risk assessment, and patient and public involvement, we propose a necessary framework for sustainable ethical innovation in healthcare technology. The success of this field will depend on interdisciplinary cooperation and the advance in sustainable ethic innovation.

**Systematic Review Registration:**

PROSPERO, identifier CRD42019134313.

## Introduction

The global health challenge of dementia is exceptional in size, cost and impact (Wortmann, 2012). The World Health Organization estimates that 47 million persons live with dementia worldwide, a number expected to reach 75 million by 2030 and more than triple by 2045 (World Health Organization, 2017). According to the Alzheimer’s Association, dementia-related costs range from $157 to $215 billion - higher than costs associated with cancer or cardiac disease — in the US alone, with roughly $42,000 to $56,000 spent per individual. These costs are driven to a significant extent by behavioral and psychological symptoms of dementia (BPSD) such as psychosis, apathy, hyperactivity, agitation, sleep disorders or depression (Ballard and Howard, 2006). This symptomatology may be caused or exaggerated by a range of conditions, such as hypoglycemia, pain and general discomfort, or they may arise secondary to the use of both psychotropic and non-psychotropic medications, which are known to precipitate a wide range of symptoms (Lyketsos et al., 2006). The prevalence of polypharmacy further adds to this clinical challenge (Gulla et al., 2016). Compounding this, no FDA approved pharmacologic treatments for BPSD exist and a wide range of psychotropic medications — including antipsychotics, mood stabilizers, antidepressants, and cholinesterase inhibitors — are regularly used to manage the symptoms, despite clear guidelines as to when and how to use them (Ballard and Corbett, 2010). This has led to vast variance in clinical practice around pharmacologic management of BPSD (Livingston et al., 2017). Polypharmacy and inappropriate prescribing can lead to significant adverse events, including increased fall risk, higher rates of inpatient care, loss of independence, and it increase the need for monitoring, which can significantly raise costs of dementia care, especially in nursing homes (Winblad et al., 2016).

Thus, there is an urgent need for tools that facilitate diagnoses that are more precise and a deeper understanding of patterns and triggers for BPSD (Kang et al., 2010). This includes tools that generate continuous data on behavior patterns, which may facilitate earlier detection of temporal events and guide more precise pharmacotherapy. Finally, there is a need for tools that can more closely monitor treatment response in dementia across care settings (Teipel et al., 2018).

A wide array of new technologies may provide solutions, especially those explicitly designed to support people with dementia and their formal and informal caregivers (Yang and Kels, 2017). The evidence around this has also been growing with research highlighting aspects of active and passive technology used in dementia (Pillai and Bonner-Jackson, 2015; Martinez-Alcala et al., 2016; Giggins et al., 2017; Brims and Oliver, 2018), the impact of safety equipment on wandering in dementia (Lin et al., 2014; Mangini and Wick, 2017), ethical considerations of surveillance technology in dementia (Sorell and Draper, 2012), or the need for real-world evidence-based solutions to conduct clinical trials (Teipel et al., 2018).

In this review paper, we present a synopsis of existing research studies in this space, including work on both commercially available as well as prototype technologies. This includes diagnostic technologies that utilize active and passive sensing in connection with smart housing, voice recognition and motion mapping (Teipel et al., 2018), and prognostic approaches that may inform clinicians about a range of potential responses, including alterations in circadian rhythm, changes in gait speed, falls, and variations in spatial location and reduction in resistance to care.

Finally, we discuss the potential pitfalls of this technology, specifically related to issues around ethics, privacy and security of data (Bantry-White, 2018; Chalghoumi et al., 2019) and the scalability of these technologies in terms to social living and activities.

## Methods

This systematic review presents a synthesis of previous research on sensing technology to assess behavioral and psychological symptoms and to monitor treatment response in people with dementia.

### Literature Search

We initially searched for peer-reviewed English language publications indexed in the following databases: Embase, Medline, Cochrane library and Web of Sciences, published up to the 5^th^of April 2019. Keywords included MESH terms and phrases synonymous with “dementia”, “sensor”, “patient”, “monitoring”, “behavior”, “therapy”. See full search history in the supplementary material. We assessed papers for eligibility using the PICO criteria (P: population, I: intervention, C: comparison and O: outcome), (see **Table 1**). We included studies applying both cross-sectional and prospective designs, including randomized controlled trials, cohort studies, and case-control studies. Reviews, opinion papers, protocols, and conference abstracts were excluded from the main search results.

**Table 1:** Inclusion and exclusion criteria.

Inclusion criteria according to PICO	Population	People with dementia
	Intervention	Use of sensor technology
	Comparison	No use of sensor technology
	Outcome	Changes in behavioral and psychological symptoms in dementia/neuropsychiatric symptoms in dementia. Validity of assessment of neuropsychiatric symptoms in dementia comparing sensor technology with proxy rated symptoms
**Exclusion criteria**	Studies published before 2009. Reviews, protocols, opinion, and conference papers. Publications in other languages than English.	

After removal of duplicates, one researcher (BH) screened all the manuscripts on title and abstract level to select relevant studies based on the inclusion and exclusion criteria. Potentially relevant studies were assessed for eligibility by all coauthors by evaluating the inclusion and exclusion criteria on the full-text manuscripts. Reference lists of manuscripts and reviews were screened to identify additional relevant publications. The final selection of included publications was by consensus among all authors. The study was registered in PROSPERO 3^rd^ of May 2019.

## Results

**Figure 1:**
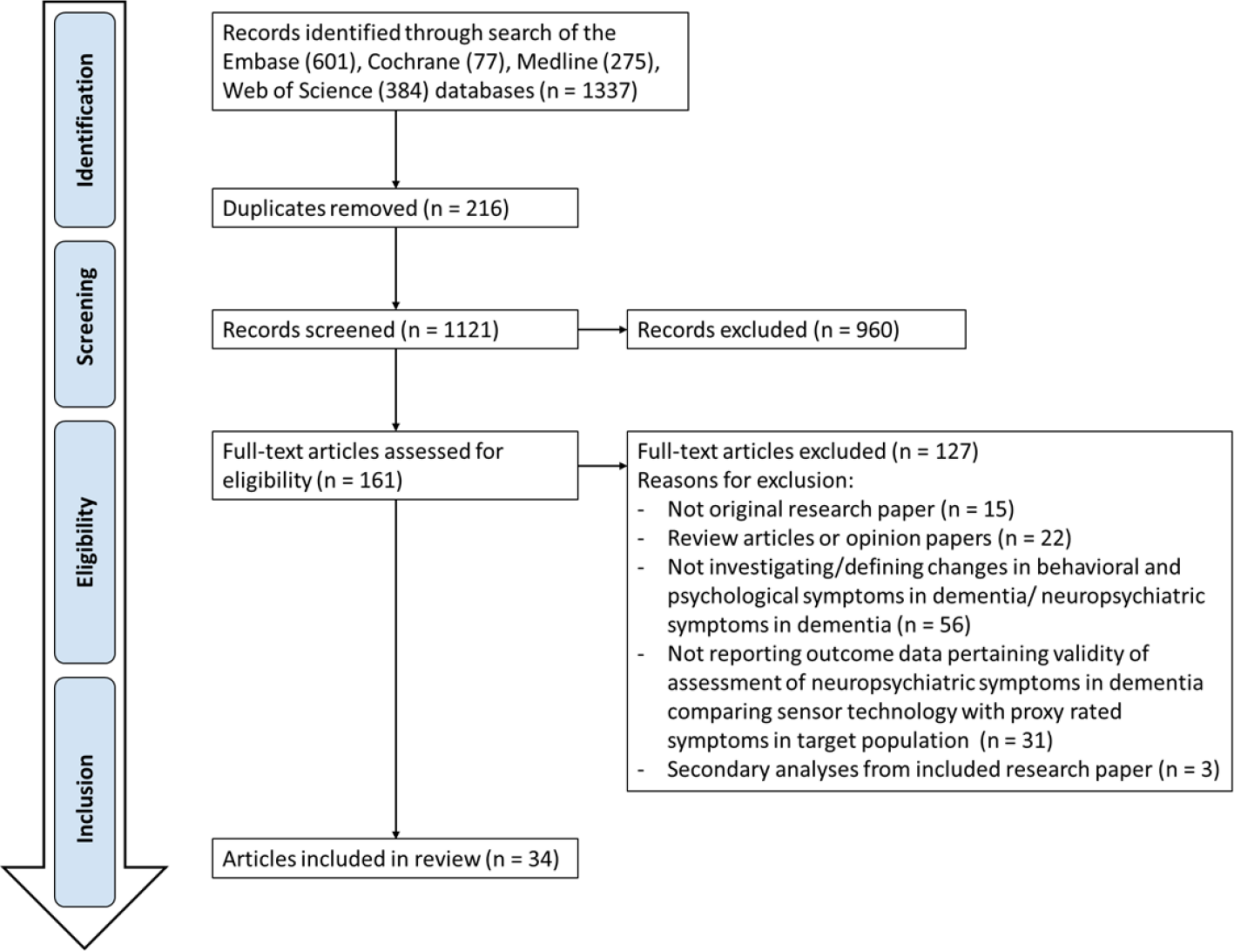
Flow Chart.

The systematic search generated 1,337 potential publications from Embase (601), Cochrane (77) Medline (275), and Web of Science (384), of which 161 papers were identified as relevant for full-text evaluation (**Figure 1**). Of these, 127 papers were removed from the following results because they were either editorial review pieces or opinion articles, or because the studies involved use of technology but not for the primary goal of managing BPSD. Eighteen (53%) articles were published in Europe, 10 (29%) in the United States and 6 (18%) in Oceania.

Of the 34 studies selected, 23 focused on management of BPSD itself, while 11 studies that utilized sophisticated technological approaches for studying other factors. For example, one study (Whelan et al., 2018) assessed communication between caregivers and nursing home staff, while another utilized technology to assess ability to perform activities of daily living (ADLs) (Stucki et al., 2014). While these studies did not meet the original inclusion criteria, by consensus among the co-authors, we elected to include the studies since they reflect potentially meaningful applications of technology and may have implications for pharmacologic and non-pharmacologic management of BPSD.

For our final review, we assessed the full text from the 34 papers that were identified as relevant and divided studies into four broad categories, based on the type of technology used: (1) wearable technology, (2) non-wearable motion sensor technology, (3) assistive technology/smart home technology, and (4) other technologies not meeting criteria for the above three. We identified six papers that utilized more than one type of technology, and incorporated them into one of the above sections, based on the primary technology for each study.

### Wearable Technologies

We identified seven studies that used wearable technologies — these included multiple sensor systems (two papers), ankle or wristbands (three papers), or a combination of both (two papers). We identified four prospective or retrospective cross-sectional studies along with two cohort studies and one case-control study. Study length ranged from 100 sec to 18 months. We noted that five studies utilized wearable technologies to primarily detect motion and two papers utilized wearable technologies that detected variables other than motion (body posture, stress). The details of the identified studies are summarized in **Table 2**.

**Table 2:** Studies utilizing wearable technologies.

Author Country	Year	Study design	N	Study length	Domains studied	Outcome measures	Type of technology
Bankole et al., USA (Bankole et al., 2012)	2012	Cross-sectional	6	6 weeks	Agitation in dementia	Construct validity of BSN, tested against CMAI, ABS, MMSE	BSN - readings from wearables on wrist, waist, and ankle
Fleiner et al., Germany, (Fleiner et al., 2016)	2016	Cross-sectional	45	72 h	Agitation in dementia	Feasibility and acceptance of wearable uSense sensor	Wearable “uSense” 3D hybrid motion sensor on lower back which records body postures
Hsu et al. Taiwan, (Hsu et al., 2014)	2014	Cross-sectional	71	1 visit	Dementia	Validity of wearable device in sensing gait and balance problems during walking tasks	Inertial sensor-based wearable
Kikhia et al. Sweden, (Kikhia et al., 2016)	2016	Case series	6	37 days	Stress in dementia	Stress measurements (data was categorized into Sleeping, Aggression, Stress, and Normal) and GSR data	Wearable (“DemaWare@NH” wristband) - includes accelerometer, detects skin conductance and temperature, and environmental light and temperature
Merilahti et al. Finland, (Merilahti et al., 2016)	2016	Retrospective database study	16	12–18 months	Sleep patterns and functional status	Actigraphy, ADLs	Wearable (wristband)
Zhou et al. USA, (Zhou et al., 2017)	2017	Cohort study	30	1 visit	Motor-cognitive impairment	Feasibility of iTMT, performance on iTMT	iTMT
Zhou et al. USA, (Zhou et al., 2018)	2018	Cohort study	44	1 visit	Cognitive frailty (cognitive impairment and frailty)	Gait, iTMT performance, accuracy of iTMT system in detecting motor planning errors	iTMT

ABS, Aggressive Behavior Scale; ADL, Activities of Daily Living; BSN, Body sensor network; CMAI, Cohen-Mansfield Agitation Inventory; GSR, Galvanic Skin Response; iTMT, Instrumented trail-making task; MMSE, Mini-Mental-Scale Examination.

### Non-Wearable Motion Sensor Technologies

We identified 12 studies that utilized sensor-based motion detection approaches other than wearables. Only one of these was a randomized controlled trial, with one cohort study, four cross-sectional studies, two proof of concept studies, two case studies, and one longitudinal study. The sample sizes of the studies ranged vastly — from 1 to 265. This broad range reflects the heterogeneity of applications of motion sensor technologies for dementia. Study length ranged from two weeks to three years. Likewise, in the case of wearable technologies, we identified vast heterogeneity in study indications and purpose. Identified studies are summarized in **Table 3**.

**Table 3:** Studies utilizing motion-sensing technologies.

Author Country	Year	Study design	N	Study length	Domains Studied	Outcome measures	Type of technology
Akl et al., Canada, (Akl et al., 2015)	2015	Feasibility Study	97	3 years	Mild cognitive impairment	CDR), MMSE (tracking who remained cognitively intact vs. who experienced decline)	Passive infrared motion sensors, wireless contact switches (to track entrances/exits), and motion-activated sensors (to track walking speeds) installed in the home, machine learning algorithms
Alvarez et al. Spain (Alvarez et al., 2018)	2018	Cohort Study	18	10 weeks	Freezing of gait & abnormal motion behavior	Accuracy of Measurements	Multisensory band (wearable - temp, HR, motion data), binary sensor (doors open/close), RGB-D camera (extraction of depth information), Zenith camera (360-degree pano camera for movement tracking), WSN anchors/beacons (monitor signals from pts' wearables)
Dodge et al. USA, (Dodge et al., 2015)	2015	longitudinal	265	3 years	MCI	CDR; Neuropsychiatric scales (immediate & delayed recall; category fluency; trails, WAIS, Boston Naming Test)	Passive in-home sensor technology (specific motion sensors on the ceiling)
Enshaeifar et al. UK (Enshaeifar et al., 2018)	2018	Cross-sectional	12	6 month	Dementia (agitation, irritation, and aggression)	Motion data and level of engagement in activities	Wireless sensors (passive infrared sensors, motion sensors, pressure sensor, central energy consumption monitoring device)
Galambos et al. USA (Galambos et al., 2013)	2013	Case Series	5	7–12 months	Depression & Dementia in older adults	Congruence between health information (GDS, MMSE, SF-12) and activity level changes	Passive infrared motion sensors
Gochoo et al. USA (Gochoo et al., 2018)	2018	Cross-sectional	1	21 months	Dementia	Accuracy of classifier model in correlating travel pattern with dementia detection	Passive Infrared sensors & deep convolutional neural network (DCNN)
Jansen et al. Germany, (Jansen et al., 2017)	2017	Cross-sectional	65	2 consecutive days	motor & cognitive impairment in adults in 2 nursing homes (motion, gait, cognitive function)	MMSE, GDS, apathy evaluation scale, short falls efficacy scale international, movement tracking (time away from room, transits)	Wireless sensor network (nodes fixed to the walls that use radio signals)
Melander et al. Sweden (Melander et al., 2017)	2017	Feasibility & Observational	9	2 weeks	Dementia, agitation	Correlational analysis	EDA Sensor
Melander et al. Sweden, (Melander et al., 2018)	2018	Case Series	14	8 week study duration	Dementia, agitation	NPI-NH (Nursing home), Electro dermal activity (EDA)	EDA Sensor
Nishikata et al. Japan, (Nishikata et al., 2013)	2013	Cross-sectional	40	21-191 days	Moderate to advanced AD; BPSD		Integrated Circuit tag monitoring system (antennas set up on the ceiling that receive signals when patients moved under them)
Rowe et al. USA (Rowe et al., 2009)	2019	RCT	106	12 months	Nighttime wandering in dementia	Feasibility of system; prevention of dangerous nighttime events	Nighttime monitoring system
Yamakawa et al. Japan (Yamakawa et al., 2012)	2012	Cross-sectional	35	95 days	Nighttime wandering in dementia	Movement indicators (distance moved, number of hours with movement, etc.), agreement with nursing records; system data agreement with BPSD measured by NPI	Integrated circuit (IC) tag monitoring system - measures temporal and spatial movements

BPSD, Behavioral and Psychological Symptoms of Dementia; CDR, Clinical Dementia Rating scale; DCNN, Deep Convolutional Neural Network; EDA, Electro Dermal Activity; GDS, Geriatric Depression Scale; HR, Human Resources; IC, Integrated circuit; MMSE, Mini-Mental-State Examination; NPI, Neuropsychiatric Inventory; NPI-NH, Neuropsychiatric Inventory-Nursing Home version; SF-12, Short-Form Health Survey 12; WSN, Wireless Sensor Network.

### Assistive/Smart Home Technologies

We identified 12 studies that utilized sensor rays placed in the living environment of study subjects. These were variously referred to as assistive or smart home technologies, since they required minimal active engagement by the patient or subject. The studies identified in this category included only one partial RCT — this study had 3 sites, but investigators were able to implement the RCT design at only one site. In addition, we identified three cross sectional/case-control studies, one cohort study, two case series and two open feasibility studies. We also identified three qualitative studies in this category. Study length ranged from 30 min interviews to 15 months and sample sizes ranged from four individuals to 65. Details of studies using these technologies are summarized in **Table 4**.

**Table 4:** Studies utilizing assistive technologies.

Author Country	Year	Study design	N	Study length	Domains studied	Outcome measures	Type of technology
Aloulou et al. Singapore (Aloulou et al., 2013)	2013	Feasibility study	10	14 months	Wandering, falls, difficulty with ADLs	Acceptability, qualitative feedback	Ambient Assistive Living technologies (motion sensors controlled by a software)
Asghar et al. UK, (Asghar et al., 2018)	2018	Cross-sectional (questionnaire based)	327	2 months	Factors impacting use of assistive technology in people with mild dementia	Survey responses	AT included mobility supports, cognitive games, reminders or prompters, social applications, and leisure supports.
Collins ME. USA (Collins, 2018)	2018	qualitative study	8	30–45 min interviews	Alzheimer’s & related dementia	AT with ADLs	AT included Wii, iPads, iPhones, computers, medication management systems, and alarms
Hattink et al. The Netherlands (Hattink et al., 2016)	2016	RCT at Germany site, pre-test/post-test design in Belgium and Netherlands	74	8 months	In-home assistive technologies’ impact on autonomy, quality of life for both people with dementia and caregivers, sense of competence	Usefulness/user-friendliness, perceived autonomy (measured by the Mastery scale and WHOQOL), QoL (measured by QOL-AD and self-report for caregivers), caregiver competence (measured by SSCQ	Rosetta system
Jekel et al. Germany, (Jekel et al., 2016)	2016	Case-control study	21	1 day	MCI	IADL tasks, feasibility questionnaire	Assistive smart home technology
Lazarou et al. Greece (Lazarou et al., 2016)	2016	Case series	4	16 weeks	MCI/Dementia/Mild Depression	MMSE, MoCA, RBMT-delayed recall, NPI, Functional Rating Scale for Symptoms of Dementia, GDS, HDRS, Functional Cognitive Assessment Scale, Perceived Stress Scale, Beck Anxiety Scale, Trail B., Beck Depression Inventory, IADL, Rey-OCFT, Test of Everyday Attention., Map Search, Visual Elevator, Telephone Search	Smart home monitoring
Martin et al. Ireland (Martin et al., 2013)	2013	Cross-sectional	8	Varied, one patient stayed on 33 months through the lifespan of the project	Dementia	Self-report questionnaires	NOCTURNAL monitoring station
Meiland et al. The Netherlands (Meiland et al., 2014)	2014	Case series	50	15 months	Dementia	CANE, GDS, user feedback questionnaire	Monitoring and assistive ICT technologies
Nijhof et al., The Netherlands, (Nijhof et al., 2013)	2013	mixed methods (qualitative, cost analysis)	14	9 month	dementia; well-being	Feasibility, cost-saving, reduction of caregiver burden, increased independence and safety	AD life system
Olsson et al. Sweden, (Olsson et al., 2018)	2018	qualitative study (interviews about use of a technology)	8	Interview follow-up after 12 week intervention study	memory impairment due to stroke		Sensor and feedback technology
Sacco et al. France (Sacco et al., 2012),	2012	Cohort Study (prospective observational Study)	64	1 day	AD and MCI	DAS	Smart home
Stucki et al. Switzerland (Stucki et al., 2014)	2014	Feasibility	11	20 days	Focus group healthy, explorative group AD	ADL	Monitoring system

AD, Alzheimer`s Disease; ADL, Activities of Daily Living; AT, Assistive technology; CANE, Camberwill Assessment of Needs for the Elderly; DAS, Daily Activity Scenario; GDS, Geriatric Depression Scale; HDRS, Hamilton Depression Rating Scale; IADL, Instrumental Activities of Daily Living; MCI, Mild Cognitive Impairment; MMSE, Mini-Mental-State Examination; MoCA, Montreal Cognitive Assessment; NPI, Neuropsychiatric Inventory; RBMT, Rivermead Behavioral Memory Test; SSCO, Short Sense of Competence questionnaire; WHOQOL, World Health Organization Quality of Life assessment instrument; QoL, Quality of Life; QoL-AD, Quality of Life in Alzheimer’s Disease; Wii, Wii Game Console.

### Other Technologies;

In addition, we identified three studies, each of which deployed a unique technological approach that could not be classified into one of the three categories above. One feasibility study (Khosla et al., 2017) used a human-like robot to assess social and emotional responses to nonhuman caregivers. Another study utilized a suite of apps administered *via* a tablet device as a nonpharmacologic intervention for agitation in dementia (Vahia et al., 2017). We identified one study that utilized a text analysis tool to detect variance and patterns of communication between patients, staff, and caregivers (Whelan et al., 2018). The details of the studies are summarized in **Table 5**.

**Table 5:** Studies utilizing other technologies.

Author Country	Year	Study design	N	Study length	Domains studied	Outcome measures	Type of technology
Khosla et al. Australia, (Khosla et al., 2017)	2016	Longitudinal	115	3 years	Social engagement in dementia	Emotional engagement, Visual engagement, Behavioral engagement, Verbal engagement, Robot acceptability questionnaire, Anxiety questionnaire	Social human robot named “Matilda”
Vahia et al. USA, (Vahia et al., 2017)	2016	Feasibility	36	Duration of hospitalization	Agitation in dementia	Acceptability, staff report of agitation severity	iPads with 70 installed applications
Whelan et al. Australia (Whelan et al., 2018)	2017	Cross-sectional	34	10-min conversations	Communication difficulties between people with dementia and caregivers (e.g., topic shifts, interference, non-specificity, etc.)	Validity of Discursis software in detecting different types of “trouble-indicating behaviors” when checked against human coding	Discursis software (automated text-analytic tool which quantifies communication behavior

Finally, during the entire review process, we became increasingly aware of the discrepancies and lack of consensus of the terminology used in this field. An overview of terminology and content are presented in **Table 6**.

**Table 6:** Terminology and content of different devices.

Terms	Devices	Tasks
Noninvasive body sensor network technology	Wearables on wrist, waist, and ankle e.g. accelerometer	Detect skin constitution; skin temperature; activities; environmental light and temperature
3D Hybrid motion sensors of body postures	Uni- and multi-axial accelerometers	Body posture
Unobtrusive sensing technologies with signal processing of real-world data (or monitoring system (TIHM) using Internet of Things, IoT)	Passive, wireless infrared motion sensors, analyzed by machine learning algorithms	Tracks entrances/exits and walking speeds in the home Track motion; pressure; central energy consumption
Integrated Circuit tag monitoring system	Antennas set up on the ceiling and related to a software platform	Register signals when patients moved under them
Passive, web-based, non-intrusive, proxy-free, assistive technology (AT)	Wii (Nintendo); iPads; iPhones; computers; video cameras; medication management, and alarms	Support of mobility and leisure; cognitive games; social robots; reminders or prompters; social applications, detection/classification of ADL/IADL deficits
Sensor and feedback technology	Individually pre-recorded voice reminder	Memory support
Information and communication technology (ICT)	Imaging and video processing to improve assessments	Detect functional impairment and be more pragmatic, ecological and objective to improve prediction of future dementia
Tablet devices as novel non-pharmacologic tools	iPads	70 installed applications support challenging patient behavior
Discourse analysis software	Automated text-analytic tool	Quantify communication behavior by discriminating between diverse types of trouble and repair signalling behavior

## Discussion

The goal for this review was to identify and summarize the extent to which literature on technologies (specifically sensors) have been used in the assessment and management of behavioral and psychological symptoms of dementia. As these technologies become widely available, this role is likely to expand (Collier et al., 2018). We identified several ways in which these technologies are being studied. This body of literature will play a crucial role in helping researchers, clinicians and municipalities, and industry partners to develop precision approaches to dementia care. We did note, however, that even though we in our original search aimed at clinical intervention studies with control groups, the majority of the studies found are preliminary with relatively small sample sizes and small durations. Some studies with much larger sample sizes were not intervention studies; rather they represented large surveys of participants around technology use. This dearth in intervention studies suggests that the grounds for innovation, validation, and clinical transference of technology in the management of behavioral symptoms are fertile.

Though we classified technologies into three broad categories, we identified several common underlying themes. Firstly, almost half of studies across the three categories represent ways in which temporarily dense data on motion can be processed and aggregated as proxy for behavior. Findings from these studies indicate high validity of using motion data to detect and track behavioral symptoms such as sleep disturbances, agitation, and wandering (Rowe et al., 2009; Bankole et al., 2012; Sacco et al., 2012; Yamakawa et al., 2012; Aloulou et al., 2013; Galambos et al., 2013; Stucki et al., 2014; Fleiner et al., 2016; Hattink et al., 2016; Jekel et al., 2016; Lazarou et al., 2016; Merilahti et al., 2016; Alvarez et al., 2018; Enshaeifar et al., 2018). Continuous motion monitoring of people with dementia using sensor technology provides informal caregivers and health care providers with the ability to more immediately and accurately diagnose and manage behavioral disturbances and can help to delay admission to long-term care or inpatient facilities. In a prodromal population, data from 8 of the studies suggest that motion data can also be useful in early detection of mild cognitive impairment and/or mild Alzheimer`s disease (Sacco et al., 2012; Hsu et al., 2014; Akl et al., 2015; Dodge et al., 2015; Gochoo et al., 2018; Zhou et al., 2018). While the majority of identified studies focused on the assessment of behaviors, we also identified 8 studies that developed intervention approaches based on sensor data or other feedback (Rowe et al., 2009; Aloulou et al., 2013; Martin et al., 2013; Hattink et al., 2016; Vahia et al., 2017; Khosla et al., 2017; Melander et al., 2018).

In addition, out of 34 studies, we found that 16 studies represented proof-of-concept, acceptability, and/or feasibility testing for technologies that are new and have not been used in the dementia population previously. These studies demonstrated some usability issues for smart home and assistive systems, e.g., technological malfunctions and general user-unfriendliness; however, the technology used was predominantly non-intrusive and well-accepted (Hattink et al., 2016; Olsson et al., 2018).

In terms of data privacy and security, we noted that the majority of our identified articles conclude their discussion by encouraging stakeholders to respect users’ privacy and autonomy. Several ask for legal frameworks and regulations to monitor the rapid development of this promising area (Yokokawa, 2012; Yang and Kels, 2017; Khan et al., 2018; Teipel et al., 2018). While we did not specifically identify clinical studies related to ethics, data privacy and security in our review, we present a synopsis of this topic, since the eventual acceptability of new technologies in dementia will be contingent on the development of transparency and trust around digital tools. This is highlighted in several opinion papers and review articles, which discuss ethical considerations in sensing technology for people with dementia or intellectual and developmental disabilities (**Table 7**).

**Table 7:** Review and opinion articles on ethical considerations in sensing technology for people with dementia or intellectual and developmental disabilities.

Author	Year	Type of paper	Ethical considerations
Bantry-White et al. Ireland (Bantry-White E, 2018)	2018	Scope review on ethics of electronic monitoring in PWD	a) Autonomy/liberty: Who decides the person`s interests? Identification of past and present wishes for ethical decision making; liberty by electronic monitoring; b) Privacy: Monitoring may be less intrusive than constant caregiver presence; c) Dignity: May technology be a stigma in context to a social construct? d) Monitoring formal and informal caregiving may restrict harmful behaviour. e) Beneficence/non-maleficence: Monitoring may reduce costs, but increasing isolation.
Chalghoumi et al. Canada (Chalghoumi H, et al., 2019)	2019	Focus group interviews with 6 people with I/DD	People show awareness of privacy concerns but not due to the use of technology. Privacy breaches are a major risk in I/DD: they do not understand the use of personal information and are vulnerable to biases in data collection.
Friedman et al. USA (Friedman and Rizzolo, 2017)	2017	National I/DD survey on electric video monitoring	Video monitoring are effective methods to expand community care while being cost effective. However, it should also aim at improving care, not only serve as a substitute for personal care and interaction.
Kang et al. USA (Kang et al., 2010)	2010	Opinion paper on in situ monitoring of older adults	Monitoring can replace caregiver-patient interaction and social contact but also the opposite in providing increased opportunities in contact with family members because of larger awareness of patients` needs.
Landau et al. Israel (Landau et al., 2010)	2011	Mixed method recommendations for policy makers on ethics on GPS use for PWD	a) Maintain balance between the needs of PWD for protection and safety and their need for autonomy and privacy; b) Decision for GPS use together with PWD (informed consent) and family; c) Advance directives or earlier wishes in case of lack of informed consent; d) Involvement of formal caregivers in decision making.
Mehrabian et al. Bulgaria (Mehrabian et al., 2014)	2014	Semi-structured interviews with PWD & caregivers	Participants are positive to home telecare, cognitive stimulation program and devices’ care of emergencies with potential to improve QoL. Ethical concerns (e.g. way of provision, installation, monitoring) are reported with needs for proper implementation and informed consent.
Robinson L et al. UK (Robinson et al., 2013)	2013	Scope review on practice & future direction	Summarize current use of assistive technology with focus on effectiveness, and potential benefits, and discuss the ethical issues associated with the use in elderly people including future directions.
Sorell et al., UK (Sorell and Draper, 2012)	2012	Position paper on telecare, surveillance and welfare state	Telecare may not be regarded as objectionable extension of a “surveillance state (Orwellian),” but a danger of deepening the isolation of those who use it. Telecare aims to reduce costs of public social and health care; correlative problem of isolation must be addressed alongside promoting independence.
Teipel et al. Germany (Teipel et al., 2018)	2018	Position paper on ICT devices and algorithms to monitor behavior in PWD	This paper discusses clinical, technological, ethical, regulatory, user-centred requirements for collecting continuously RWE data in RCTs. Data safety, quality, privacy and regulations need to be addressed by sensor technologies, which will provide access to user relevant outcomes and broader cohorts of participants than currently sampled in RCTs.
van Hoof et al. NL (van Hoof et al., 2018)	2018	Explorative study on RTLS in NHs	Interviews with formal caregivers; NH patients and family members, and researchers. Concerns differed between groups and addressed security, privacy of patients and carers, responsibility.
Wigg et al. USA (Wigg, 2010)	2010	Position paper on surveillance of pacing in PWD	Surveillance technologies such as locked doors dehumanise and frighten individuals, whereas motion detectors may increase QoL, health benefits and safe medication with less riskiness.
Yang et al. USA (Yang and Kels, 2017)	2017	Scope review on ethics of electronic monitoring for PWD	To protect and empower PWD, the decision-making capacity of the person has to be evaluated and a multidisciplinary process (including PWD, relatives and healthcare professionals) have to be conducted before electronic monitoring (GPS, radiofrequency, cellular triangulation) is used.

ICT, Information and Communication Technology; I/DD, Intellectual and developmental disabilities; GPS, Global Positioning System; NH, Nursing Home; PWD, People with Dementia; QoL, Quality of Life; RCT, Randomized Controlled Trial; RTLS, Real Time Location Systems; RWE, Real World Evidence.

**Figure 2:**
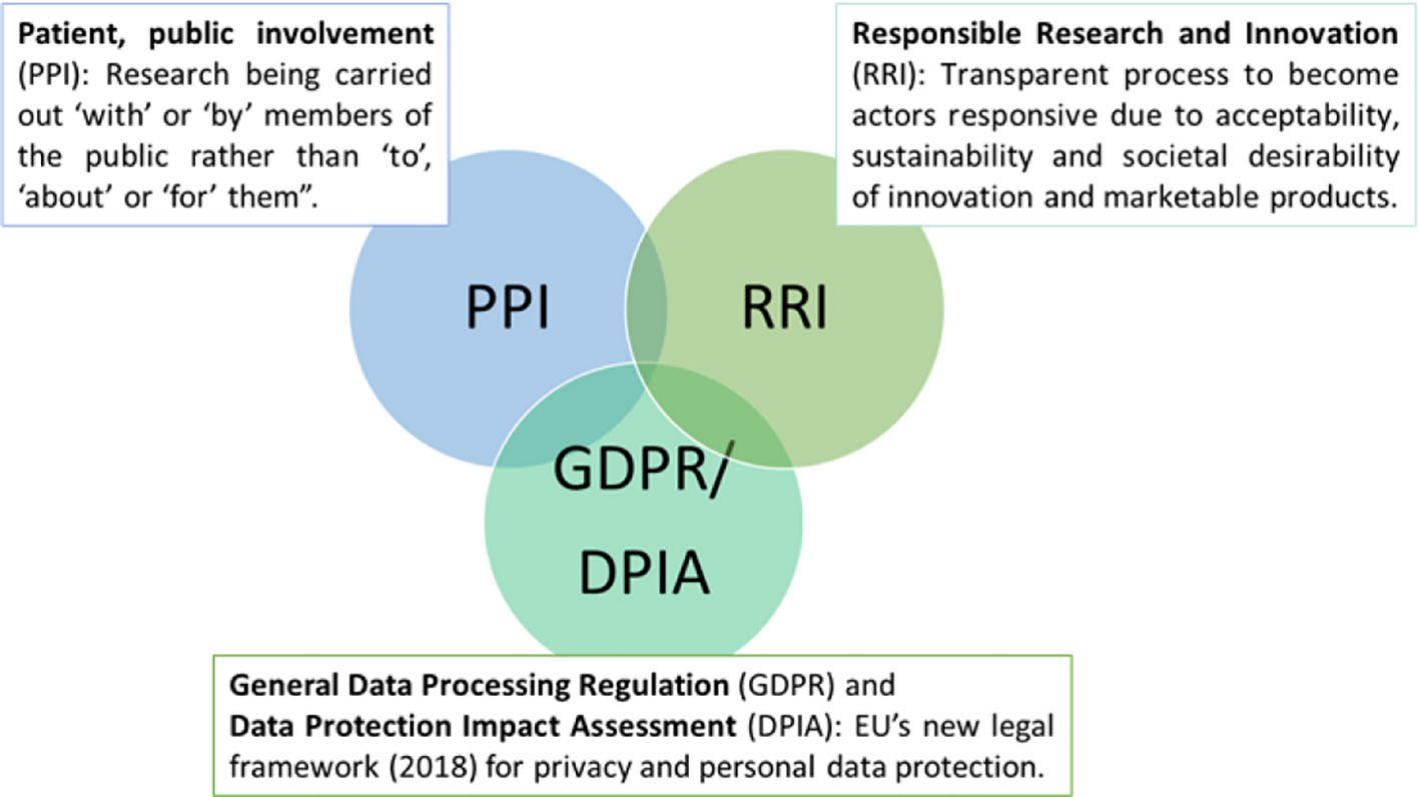
Framework for sustainable ethic in healthcare technology.

Launched in May 2018, the General Data Protection Regulation (GDPR) is the novel European Union-wide law on data protection — a significant step towards more responsible protection of individuals (Crutzen et al., 2019). While it is recognized that participation in research is based on affirmative, unambiguous, voluntary, informed, and specific consent (Mendelson, 2018), people with advanced dementia or intellectual and developmental disabilities are not able to give informed consent or understand the consequences of data acquisition (Friedman and Rizzolo, 2017; Chalghoumi et al., 2019; Timmers et al., 2019). Article 6 of the GDPR addresses this issue by including provisions that protecting persons with dementia and their relatives from being coerced into providing consent without awareness of how their data will be used (Cool, 2019; Crutzen et al., 2019). Despite this regulation, local legislation differs between European countries (de Lange et al., 2019). In Norway, for, e.g., a family member or legal advocate may provide or refuse consent based on their determination around whether the person with dementia would agree or decline to participate in a given study (Husebo et al., 2019). In Germany, the inclusion of people with dementia is limited for only those who may directly benefit from research results. To further strengthen privacy protections, Article 35 of the GDPR requires the Data Protection Impact Assessment (DPIA) (**Figure 2**) (Donnelly and McDonagh, 2019), which mandates that only the most relevant personal data is collected (data minimization), and limits data access to those who are authorized or given permission by the individual (Yang and Kels, 2017). Overall, in this review, we did not specifically include search keywords relating to ethics in sensor technology but we recognized an engaged discussion in a considerable number of position papers and reviews around ethical considerations and especially, the need for data protection, proper transfer and storage (Holm and Ploug, 2013; Ploug and Holm, 2013).

Agencies that provide funding for research increasingly require patient and public involvement (PPI) in design, implementation, and dissemination of health research (**Figure 2**) (Melander et al., 2017; Melander et al., 2018). The goal of PPI is to ensure user-centered design so that persons who may benefit from it have an opportunity, especially in the early stages of their disease (Landau and Werner, 2012; Bantry-White, 2018) to understand the purpose of the technology (e.g. GPS) and to express values, wishes, and concerns to formal and informal caregivers, We noted that this principle was incorporated into at least three studies that we reviewed (Landau and Werner, 2012; Lariviere, 2017; Mangini and Wick, 2017; Bantry-White, 2018). This approach is also likely to optimize technology engagement in dementia (Nijhof et al., 2013; Mehrabian et al., 2014). A related principle, Responsible Research and Innovation (RRI) (**Figure 2**), is defined as a transparent, interactive process by which societal actors and innovators become mutually responsive to each other (von Schomberg, 2013). They are encouraged to assume a critical perspective when evaluating the innovation and marketability of products (Holthe et al., 2018; Lehoux and Grimard, 2018). This approach may serve to promote awareness of technologies and related issues across both groups of stakeholders (van Haeften-van Dijk et al., 2015; Wu et al., 2017; Rostill et al., 2018).

## Limitations

Our findings and recommendations must be interpreted in the context of some limitations. During the process, we recognized that the MESH terms and phrases synonymous with “dementia”, “sensor”, “patient”, “monitoring”, and “behavior”, and “ therapy” probably did not cover the whole range of interesting topics. For instance, items such as ethics, activities of daily living (ADL), and communication, may increase the understanding for and connection to the clinical aspects of this quickly developing area. Because of the vast heterogeneity of the literature, including terminology and definitions of sensing technology, a meta-analysis that may facilitate aggregated recommendations was not feasible. We also noted that the majority of the studies were open-label early-stage studies. Replication of these findings in larger trials will be required before these findings can become the standard of care. Our search algorithm also has potential limitations. We restricted our search to the past decade, since we anticipate that future sensor-based care models will be built on contemporary technology. We determined that tools that are more than a decade old are unlikely to have relevance in the future.

## Conclusion

Overall, our systematic review demonstrates that sensor technologies have a broad range of potential applications in dementia care, ranging from early detection of cognitive impairment to aid in the management of behavioral and psychological symptoms in late stage dementia. Targeted clinical application of specific technologies is poised to revolutionize precision care in dementia as these technologies may be used by patients themselves, caregivers, or even applied at a systems level (e.g., nursing homes) to provide more safe and effective care. As sensor technology matures in its ability to guide care in BPSD, it may generate novel ways to capture early symptomatology (e.g., social isolation), improve specificity for cognitive testing in-situ and facilitate cost effective research approaches. A small but rapidly growing body of evidence around sensors in dementia care is paving the early way for the field, bringing into focus both the potential and pitfalls of this approach. Next step in this field may be to investigate the validity of use not only for care purposes, but also for prognostics as well as acceptability, feasibility, and responsiveness in clinical trials. The eventual success of this field will depend on inter-disciplinary models of research, development by industry partners, and sustainable ethic innovation in healthcare technology and smart housing.

## Data Availability Statement

All datasets generated for this study are included in the article/supplementary material.

## Author Contributions

BH and IV developed the study idea, designed the study protocol for the systematic search and BH applied for funding. BH screened all the manuscripts on title and abstract level to select relevant studies. All the co-authors assessed potentially relevant studies on full-text manuscripts for eligibility using inclusion and exclusion criteria. HH, LB, PO, and AR drafted the first version of the result evaluation with supervision from IV and BH. Contribution to the subsequent drafts were provided by BH, HH, LB, PO, AR, and IV. All authors approved the final version of the manuscript.

## Funding

This work is supported by the University of Bergen, The Norwegian Research Council and, in part, by the Technology and Aging Lab at McLean Hospital.

## Conflict of Interest

The authors declare that the research was conducted in the absence of any commercial or financial relationships that could be construed as a potential conflict of interest.
