# Corrigendum: Sensing Technology to Monitor Behavioral and Psychological Symptoms and to Assess Treatment Response in People With Dementia. A Systematic Review

**DOI:** 10.3389/fphar.2020.00254

**Published:** 2020-03-06

**Authors:** Bettina S. Husebo, Hannah L. Heintz, Line I. Berge, Praise Owoyemi, Aniqa T. Rahman, Ipsit V. Vahia

**Affiliations:** ^1^Department of Global Public Health and Primary Care, Centre for Elderly and Nursing Home Medicine, University of Bergen, Bergen, Norway; ^2^Department of Nursing Home Medicine, Municipality of Bergen, Bergen, Norway; ^3^Division of Geriatric Psychiatry, McLean Hospital, Belmont, MA, United States; ^4^NKS Olaviken Gerontopsychiatric Hospital, Bergen, Norway; ^5^Department of Psychiatry, Harvard Medical School, Boston, MA, United States

**Keywords:** dementia, sensoring, monitoring, behavior, therapy

In the original article, there was an error in the title. It was published as “Sensing Technology to Facilitate Behavioral and Psychological Symptoms and to Monitor Treatment Response in People With Dementia. A Systematic Review.”

The correct title should be “Sensing Technology to Monitor Behavioral and Psychological Symptoms and to Assess Treatment Response in People With Dementia. A Systematic Review.”

The authors apologize for this error and state that this does not change the scientific conclusions of the article in any way. The original article has been updated.

